# Strain histograms are equal to strain ratios in predicting malignancy in breast tumours

**DOI:** 10.1371/journal.pone.0186230

**Published:** 2017-10-26

**Authors:** Jonathan Frederik Carlsen, Caroline Ewertsen, Susanne Sletting, Maj-Lis Talman, Ilse Vejborg, Michael Bachmann Nielsen

**Affiliations:** 1 Department of Radiology, Rigshospitalet, Copenhagen University Hospital, Blegdamsvej 9, Copenhagen, Denmark; 2 Center for Fast Ultrasound imaging (CFU), Department of Electrical Engineering, Technical University of Denmark, Lyngby, Denmark; 3 Department of Pathology, Rigshospitalet, Copenhagen University Hospital, Blegdamsvej 9, Copenhagen, Denmark; Ospedale Pediatrico Bambino Gesu, ITALY

## Abstract

**Objectives:**

To assess whether strain histograms are equal to strain ratios in predicting breast tumour malignancy and to see if either could be used to upgrade Breast Imaging Reporting and Data System (BI-RADS) 3 tumours for immediate biopsy.

**Methods:**

Ninety-nine breast tumours were examined using B-mode BI-RADS scorings and strain elastography. Strain histograms and ratios were assessed, and areas- under-the-receiver-operating-characteristic-curve (AUROC) for each method calculated. In BI-RADS 3 tumours cut-offs for strain histogram and ratio values were calculated to see if some tumours could be upgraded for immediate biopsy. Linear regression was performed to evaluate the effect of tumour depth and size, and breast density on strain elastography.

**Results:**

Forty-four of 99 (44.4%) tumours were malignant. AUROC of BI-RADS, strain histograms and strain ratios were 0.949, 0.830 and 0.794 respectively. There was no significant difference between AUROCs of strain histograms and strain ratios (P = 0.405), while they were both inferior to BI-RADS scoring (P<0.001, P = 0.008). Four out of 26 BI-RADS 3 tumours were malignant. When cut-offs of 189 for strain histograms and 1.44 for strain ratios were used to upgrade BI-RADS 3 tumours, AUROCS were 0.961 (Strain histograms and BI-RADS) and 0.941 (Strain ratios and BI-RADS). None of them was significantly different from BI-RADS scoring alone (P = 0.249 and P = 0.414). Tumour size and depth, and breast density influenced neither strain histograms (P = 0.196, P = 0.115 and P = 0.321) nor strain ratios (P = 0.411, P = 0.596 and P = 0.321)

**Conclusion:**

Strain histogram analyses are reliable and easy to do in breast cancer diagnosis and perform comparably to strain ratio analyses. No significant difference in AUROCs between BI-RADS scoring and elastography combined with BI-RADS scoring was found in this study.

## Introduction

Elastography has emerged as an auspicious adjunct to B-mode ultrasound examination [1–3]. Ultrasound elastography has been intensively investigated for diagnosis of breast cancer, thyroid nodules and liver fibrosis. The diagnostic potential of elastography in breast cancer has been extensively examined, but the actual clinical benefits of performing elastography are not clear. Recent studies conclude that elastography should be used only as an adjunct to ultrasound Breast Imaging Reporting and Data System (BI-RADS)classification, but there is no consensus on how BI-RADS classifications and elastography should be combined [1, 2, 4].

Tumour stiffness is correlated with the risk of malignancy [1, 2, 5]. Both quantitative (shear-wave elastography) and qualitative methods (strain elastography) have been introduced [5]. The current study concerns strain elastography, in which the relative depiction of tissue stiffness makes visual classifications of tumour strain or semi quantifications necessary. Strain ratio calculation is the most commonly used semi quantification [6]. The ratio is calculated as the ratio between the strain in reference tissue ROIs (Region Of Interest) and the strain in breast tumour ROIs—the higher the strain ratio, the stiffer the tumour [7]. Inhomogeneous surrounding tissue may challenge the placing of reference ROIs, and if tumour and reference ROIs are placed at different depths, it may influence measurements [8, 9]. In strain histogram analysis, tumour stiffness is calculated as mean pixel colour values of a single ROI within the tumour [10]. Values range from 0 to 255 (from red over green to blue), and increasing tumour stiffness causes an increase in mean histogram values. Strain histograms are independent of reference ROIs, and are easy to perform, even if the surrounding tissue is inhomogeneous. Guidelines on elastography suggest that elastography should only be used to upgrade stiff tumours appearing benign on the B-mode examination, and consider them for biopsy, while other guidelines suggest, that elastography could be used to prevent biopsies in low-risk soft tumours [1, 4]. Most authors agree that elastography should be used mainly for reassessments of tumours with high risks of either false negative or false positive results, such as in BI-RADS 3 and 4a tumours [11,12]

In this study we hypothesized that the diagnostic accuracy of strain histograms was comparable to that of strain ratio calculations. We also evaluated if tumour size, tumour depth and breast density had any impact on the elastographic evaluations. The ultimate goal of the study was to assess whether strain elastography could be used to upgrade stiff BI-RADS 3 tumours to immediate biopsy without lowering the diagnostic accuracy.

## Material and methods

This study was approved by the Regional Ethical Committee on Medical Research (reference id: H-2-2013-FSP46). Patients gave informed oral consent before participation. Written consent was waived by the regional ethics commitee, because strain elastography was part of the examination set-up prior to the initiation of the study. Oral consent was documented in the patients’ medical records. This procedure was approved by the regional ethics committee.

### Inclusion of patients

Between August 2013 and March 2015 99 women (age ≥ 18 years, median age 53 years (mean 53.0, SD 16.5, range 18–88)) suspected of breast cancer and scheduled for biopsy with 108 breast tumours were prospectively and consecutively included. To avoid a clustering effect, only one tumour from each woman was randomly included for further analysis. This yielded 99 tumours for final evaluation. Women older than thirty years of age had mammography performed prior to the ultrasound examination. The same radiologist (SS > 15 year’s experience in breast radiology) performed all mammography-, ultrasound- and elastography examinations including all biopsies. Biopsies were performed as fine needle aspiration (14 tumours) or histological biopsy or excision (85 tumours) according to national guidelines. Triple consensus of diagnostic imaging (ultrasound and mammography), palpation and cytology was used as gold standard for all [13]. Histology was performed on formalin fixed, paraffin embedded slides, stained with hematoxylin and eosin, and evaluated in light microscope.

### Conventional ultrasound

A Hitachi Ascendus system (Hitachi, Tokyo, Japan) with an L75-probe with a bandwidth of 5–18 MHz or an L74-probe with a bandwidth of 5–13 MHz was used for all ultrasound examinations. BI-RADS classifications were performed according to the American College of Radiology [14]. The BI-RADS system classifies tumours as *1* (negative), *2* (benign), *3* (probably benign), *4* (suspicious) and *5* (highly suspicious of malignancy). According to national guidelines all BI-RADS 3 (probably benign), 4 (low to high suspicion of malignancy) and 5 (highly suggestive of malignancy) tumours were biopsied [15]. Some patients with BI-RADS 2 (benign) findings on ultrasound were also biopsied according to national guidelines, either due to indeterminate or suspicious findings on mammography, due to a palpable tumour or because of patient request. Two BI-RADS 1 patients were biopsied due to patient request. Histology in both cases showed non-tumour like fibrotic areas. The maximal diameter of tumours and the distance from the transducer to the tumour centre were recorded.

### Ultrasound elastography

**Fig 1:**
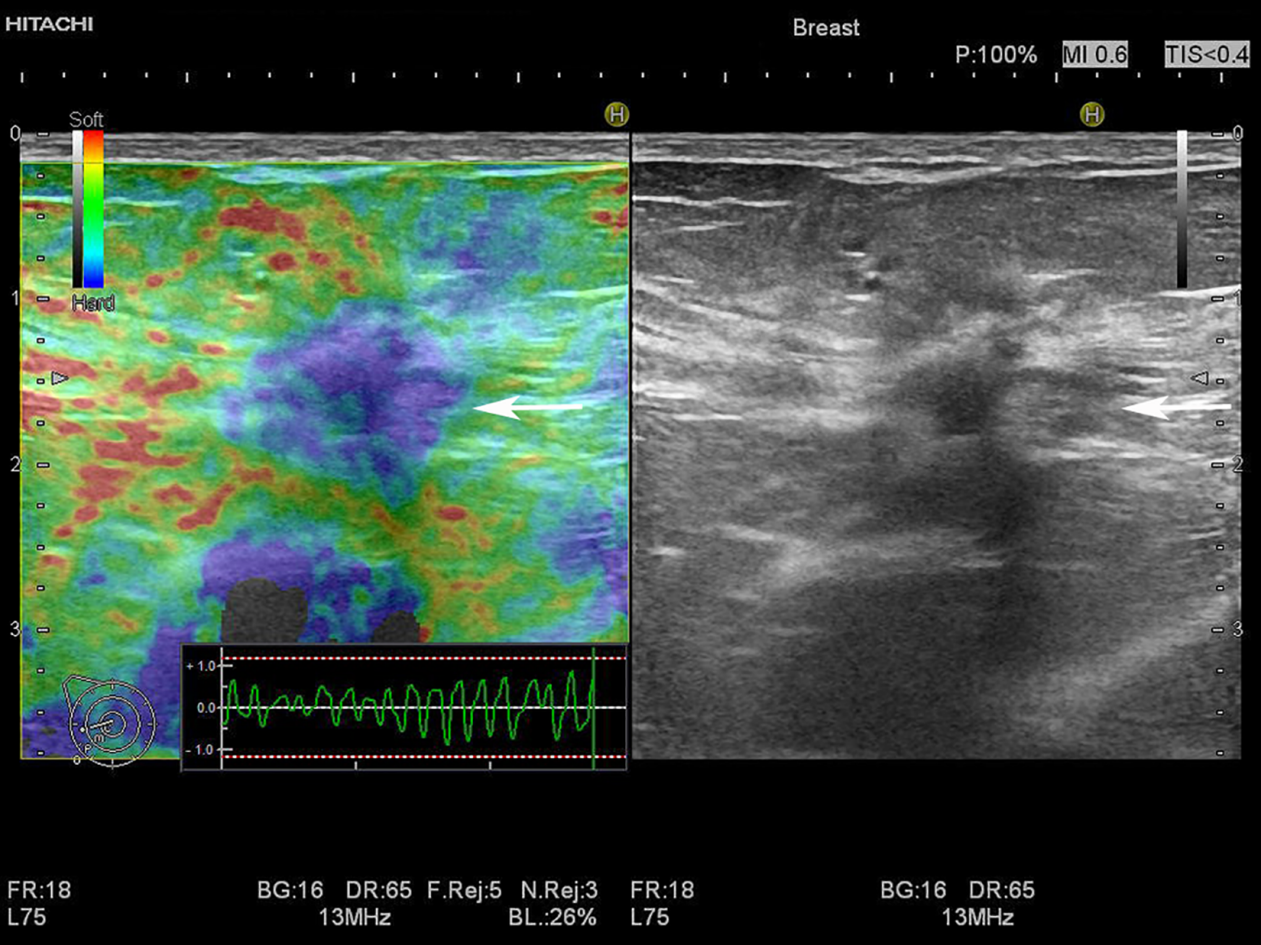
Strain elastogram of a 15 mm BI-RADS 5 tumour. The strain elastogram is displayed left of the B-mode image and shows a stiff tumour appearing mainly blue with small green areas (horizontal arrow). The tumour appears larger in the strain elastogram than in the B-mode image, indicating malignancy. The green curve on the strain quality indicator (lower right corner of the elastogram) displays the applied compression and decompression and should ideally be between the two dotted lines (red/white). The colour scale (upper left corner of the elastogram) shows the range of colours used to designate soft (red), intermediate (yellow and green) and stiff (blue) tissue. The B-mode image shows an irregular, spiculated, hypoechoic mass with posterior shadowing (horizontal arrow). Histology showed carcinoma.

Ultrasound elastography was performed in conjunction with the B-mode examination before biopsy (Fig 1). A standard breast pre-set was used, and elastograms were displayed alongside the B-mode image using a colour scale ranging from red (soft) through yellow and green to blue (stiff). The on-screen quality indicator was used to assess if transducer pressure amplitude and frequency were adequate (Fig 1). Strain histograms and ratios were assessed retrospectively. Strain ratios were calculated from still frames and strain histograms were performed on cine loops of ten seconds.

**Fig 2:**
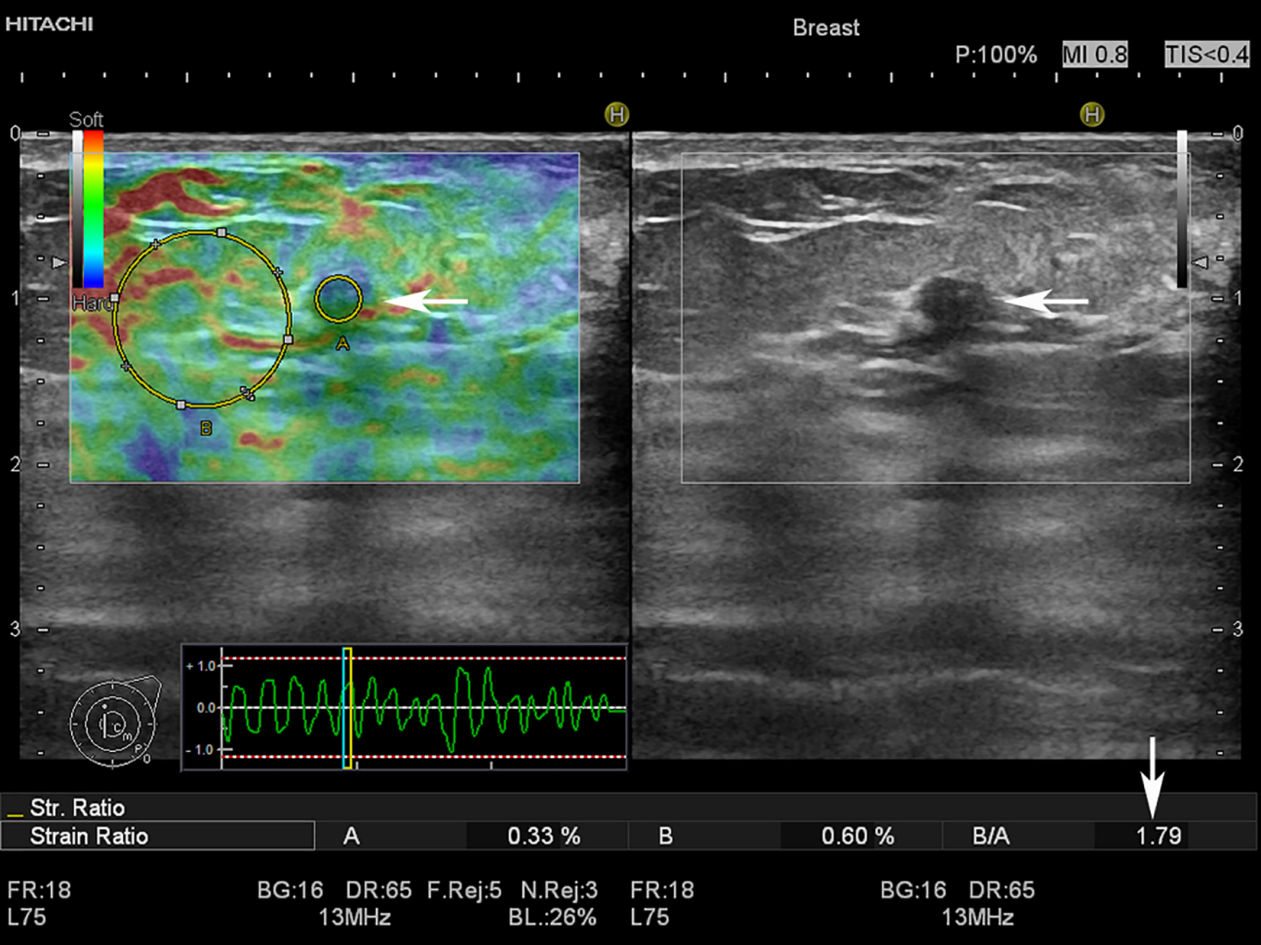
Strain ratio calculation of a 5 mm BI-RADS 3 tumour. The strain elastogram shows a green and blue tumour (horizontal arrow) indicating low suspicion of malignancy. ROI A is placed within the tumour and ROI B is placed in adjacent fatty tissue. The strain ratio is calculated as: ***average strain***_**ROI B**_ **/ *average strain***_**ROI A.**_The higher the strain ratio, the stiffer the tumour and the higher the risk of malignancy. The strain ratio, displayed in the lower right corner of the B-mode image (vertical arrow), was 1.79 signifying a relatively soft tumour. On the B-mode image, the tumour (horizontal arrow) appears oval, circumscribed, hypoechoic and wider than tall with no posterior features. Histology showed fibroadenoma.

Strain ratios were calculated using the scanner software (Fig 2). Tumour and reference ROIs were placed at the same depth, and the ratio was calculated automatically. Reference ROIs were preferably placed in fat tissue, if impossible it was placed in glandular tissue. If multiple strain ratios were calculated, the average was used for final evaluation.

**Fig 3:**
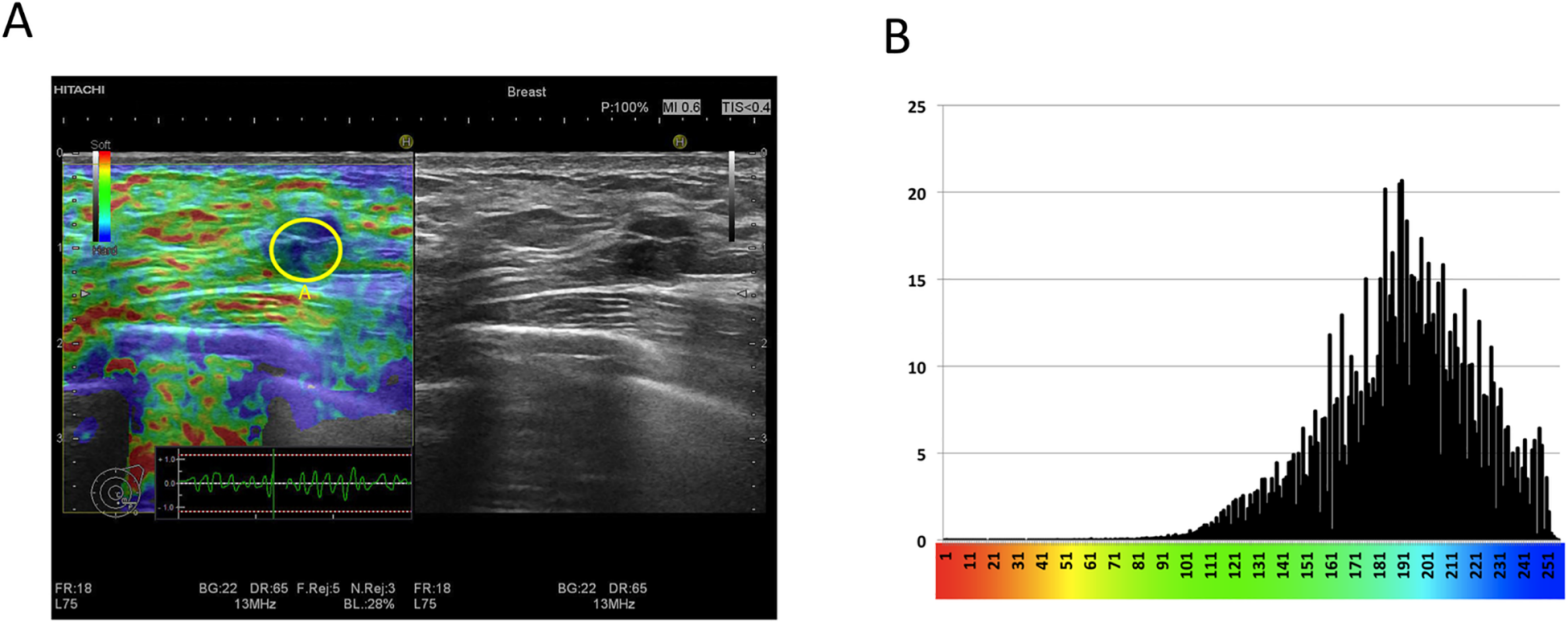
**(A)** Strain histogram analysis of an 11 mm BI-RADS 3 tumour. The strain elastogram shows a predominantly blue tumour. Strain histogram analysis was performed from a single ROI within the tumour (ROI **A**). The B-mode image shows an oval, circumscribed, hypoechoic tumour, which is wider than tall and with slight posterior enhancement. Histology showed carcinoma. **(B)** The strain histogram is displayed as a bar chart, with the pixel colour values on the x-axis, and the number of pixels of a certain colour pr. 1000 pixels within the ROI on the y-axis. The mean colour value of the tumour is then calculated. In this case the mean colour value was 190, signifying a relatively stiff tumour, consistent with carcinoma.

Strain histograms were performed off-line using the software ImageJ with a special plug-in (Fig 3) [16]. Tumour ROIs were similar to the ROIs used for strain ratio calculations. Histograms of the colour distribution within tumour ROIs were then performed on the entire cine-loop, and a mean colour value for each tumour was calculated.

### Combinations of B-mode BI-RADS and elastography

All BI-RADS 3 tumours were evaluated to see if strain histograms and ratios could potentially be used to upgrade stiff tumours to immediate biopsy. For both strain histograms and strain ratios a cut-off just below the lowest value of the softest malignant tumour was found, and all tumours above this cut-off were reclassified as BI-RADS 4. All tumours below the cut-off remained BI-RADS 3. All other BI-RADS scorings were left unchanged.

### Mammography

Most patients had mammography performed on the same day, prior to the ultrasound examination. For patients who did not have mammography performed on the same day as the biopsy, mammograms up to six months old were used for breast density evaluation. Breast density was assessed by visual inspection according to the American College of Radiology BI-RADS Atlas 4^th^ edition [14].

### Statistical analysis

Descriptive statistics were calculated using Microsoft Excel version 2011 (Redmond, WA, USA). The statistical software R version 0.98.1091 (© 2009–2014, the R Foundation, Vienna, Austria) was used for all other statistical analyses. Receiver-operator-characteristics (ROC curves) were done in SPSS version 20 (SPSS, IBM, Chicago, USA). In case of skewness in the distribution of data, data were log-transformed. Averages are presented as median and/or mean (+/- SD) and a range if relevant. Differences between means were assessed using an unpaired t-test. For evaluation of diagnostic performance, area-under-receiver-operator-characteristics (AUROC) curves were performed. For BI-RADS scoring sensitivity, specificity, positive predictive value and negative predictive value was calculated using a cut-off between 3 and 4. To assess the impact of tumour depth and tumour diameter on strain ratios and strain histograms, linear regression with backwards elimination of insignificant covariates was performed. To assess the impact of breast density, analysis of variance was performed. The significance level was set at 0.05.

## Results

Fifty-five of ninety-nine tumours were benign (median diameter 11 mm (mean: 12.29; standard deviation: 2.0; range: 4–50)) and 44 were malignant (median diameter 12 mm (mean: 11.92; standard deviation: 1.67; range: 4–42)). Histological diagnoses are summarized in Table 1. The median diameter of all 99 tumours was 12 mm (mean: 12.3; standard deviation: 1.86; range: 4–50). The distribution of tumours within the ultrasound BI-RADS classes is shown in Table 2. Mammography was performed in 91 patients, while 8 women under the age of 30 did not have mammography performed. The mean percentage mammographic density score according to BI-RADS classification for the 91 patients was 2.15 (standard deviation: 1.1).

**Table 1:** Table of the histological classifications of the included tumours.

Histology	N
**Benign tumours**	**55**
Fibroadenoma	11
Cysts	9
Fibrosis	7
Intraductal papilloma	5
Lactating glandular tissue	3
Fat Tissue	3
Fat Necrosis	3
Radial Scar	2
Inflammation	2
Ductal adenoma	2
DCIS	2
Apocrine metaplasia	2
Lymph node (benign)	1
Hemorrhage	1
Galactocele	1
Epithelial inclusion cyst	1
**Malignant tumors**	**44**
Invasive Ductal Carcinoma	40
Invasive Lobular Carcinoma	3
Myelomatosis	1

**Table 2:** Table of the distribution of malignant and benign tumours for BI-RADS 1–5.

	BI-RADS 1	BI-RADS 2	BI-RADS 3	BI-RADS 4	BI-RADS 5
**Malignant (%)**	**0 (0)**	**0 (0)**	**4 (15.38)**	**5 (45.45)**	**35 (100)**
**Benign (%)**	**2 (100)**	**25 (100)**	**22 (84.62)**	**6 (54.54)**	**0 (0)**
**Total**	**2**	**25**	**26**	**11**	**35**

The table shows the numbers of malignant, benign and total tumours (percentages in brackets) within each BI-RADS class.

**Fig 4:**
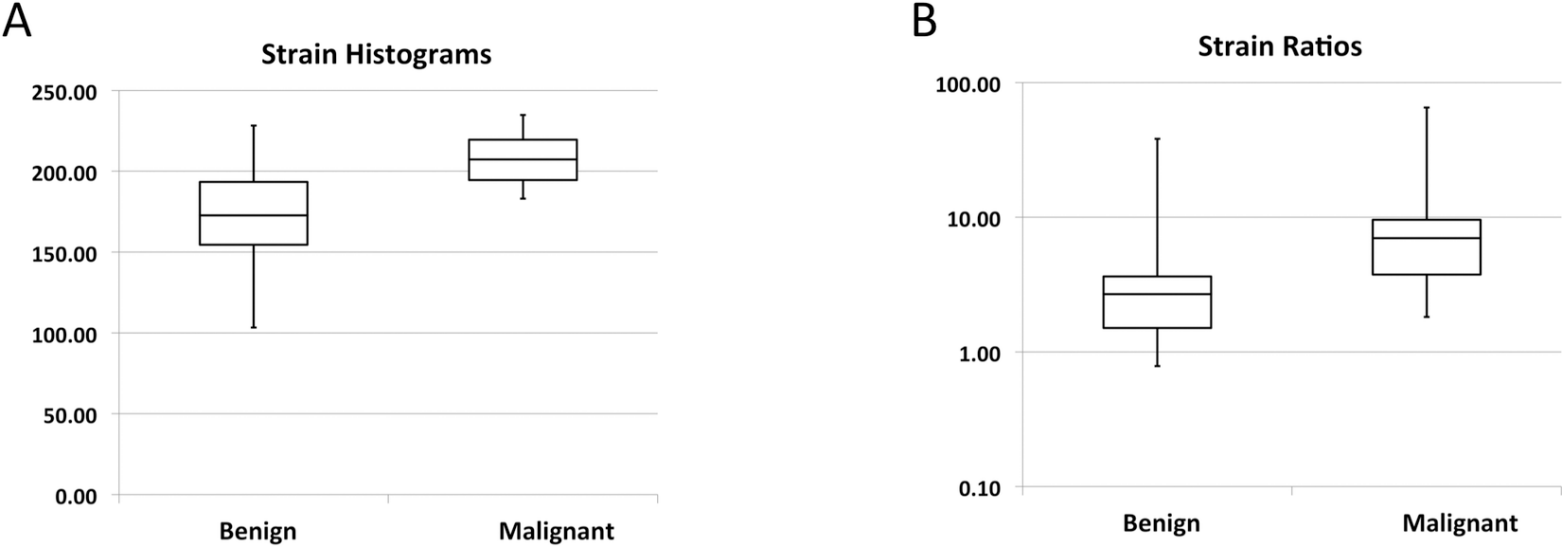
**(A)** Boxplots of the distribution of strain histogram values for benign and malignant tumours. Note that strain histogram values are unitless (y-axis, range 0–255). Upper and lower box limits are the .75 and .25 fractiles respectively. The middle box line is the average of strain histogram values. Upper and lower whiskers represent .975 and .025 fractiles respectively. **(B)** Boxplots of the distribution of strain ratio values for benign and malignant tumours. Note that strain ratio values are unitless (y-axis, range 0-∞), and that the y-axis is logarithmic. Upper and lower box limits are the .75 and .25 fractiles respectively. The middle box line is the average of strain ratios. Upper and lower whiskers represent .975 and .025 fractiles respectively.

There was a significant difference between means of benign and malignant tumours for strain histograms and strain ratios (P < 0.001 for both) (Fig 4A and 4B). For B-mode BI-RADS sensitivity was 90.9% (40/44, (CI: 77.4%–97.0%)), specificity was 89.1% (49/55, (CI: 77.1%–95.5%)), positive predictive value was 87.0% (40/46, (CI: 73.0%–94.6%)) and negative predictive value was 92.5%(49/53, CI: 80.9%–97.6%). The AUROC for B-mode BI-RADS was 0.949 (CI: 0.907–0.992). For strain histograms and strain ratios AUROCs were 0.830 (CI: 0.748–0.913) and 0.794 (CI: 0.703–0.885) respectively. The AUROC of B-mode ultrasound was significantly higher than that for both strain histograms and strain ratios (P<0.001, P = 0.008). There was no significant difference between AUROCs of strain histograms and strain ratios (P = 0.405). No effect of tumour depth, tumour maximal diameter or mammography density was seen on strain histograms (P = 0.196, P = 0.115 and P = 0.321) or on strain ratios (P = 0.411, P = 0.596 and P = 0.771).

**Fig 5:**
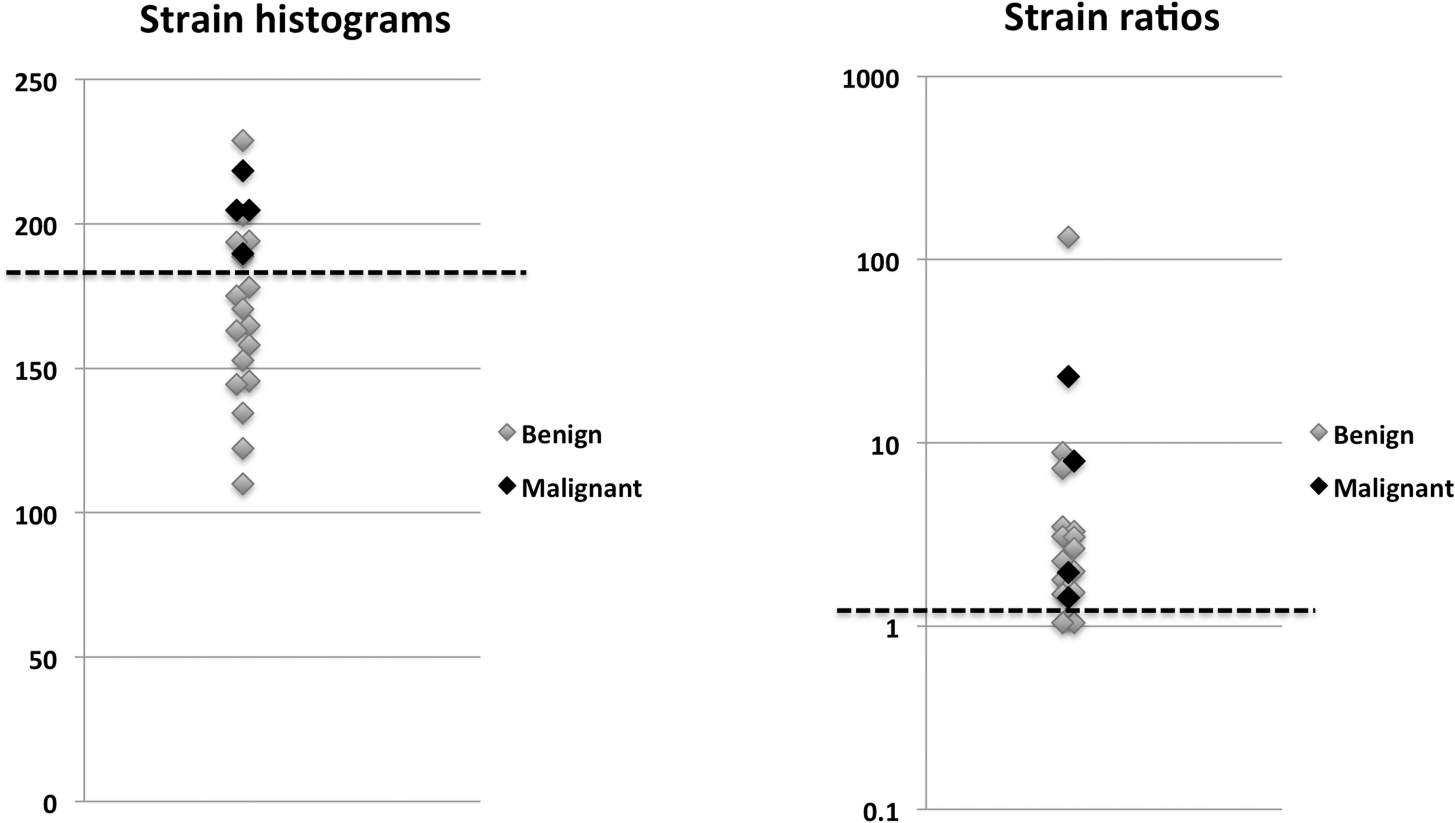
Diagrams showing the strain histogram and strain ratio values for all BI-RADS 3 tumours. Note that strain histogram values (range 0–255) are unitless, and that strain ratios (range 0-∞) are also unitless and the y-axis is logarithmic. The dashed line on each diagram shows the cut-off used when combining strain histogram (cut-off = 189) or strain ratio (cut-off = 1.44) values with ultrasound BI-RADS scoring.

Four of 26 tumours classified on ultrasound as BI-RADS 3 were malignant (Fig 5). By using a cut-off of 189 for strain histograms and 1.44 for strain ratios, all four malignant BI-RADS 3 tumours could be reclassified as BI-RADS 4, while all tumours below the cut-offs were benign. In all, ten BI-RADS 3 tumours (four malignant/six benign) were reclassified as BI-RADS 4 and immediate biopsy using strain histograms, while this was the case for 21 BI-RADS 3 tumours (four malignant/ seventeen benign) for strain ratios. This resulted in AUROCS of 0.961 (CI: 0.925–0.997) for strain histograms and BI-RADS combined and 0.941 (CI: 0.899–0.983) for strain ratios and BI-RADS combined. None of them was significantly different from BI-RADS scoring alone (P = 0.249 and P = 0.414).

## Discussion

This study evaluates the diagnostic performance of strain histograms, strain ratios and ultrasound BI-RADS scoring in a population of patients undergoing biopsy of a breast tumour. AUROCs showed that strain histograms and ratios were equal in discerning malignant from benign breast tumours, but were inferior to BI-RADS scoring.

When using strain elastography to upgrade BI-RADS 3 tumours to BI-RADS 4 and immediate biopsy, strain histograms was were able to do so with fewer benign tumours being reclassified as BI-RADS 4. The combined AUROCs of BI-RADS scoring and strain histograms and BI-RADS scoring and strain ratios were not significantly different from that of BI-RADS scoring on its own. The study was not powered to assess the different ability to successfully reclassify BI-RADS 3 tumours for immediate biopsy between strain histograms and strain ratios.

Tumour maximal diameter, depth and mammography breast density had no effect on strain histograms or ratios. Other studies have found improved diagnostic performance when combining elastography and BI-RADS-scoring [11, 17, 18]. These studies have mainly been performed with shear-wave elastography and not with strain elastography, which is still available on more different ultrasound scanner systems. Our results point towards an equal diagnostic gain in using strain elastography for reclassification of BI-RADS 3 tumours.

Few studies have investigated the application of strain histograms in breast cancer diagnosis [19–22]. In most of these studies, strain histograms were used as part of a neural network analysis, and only limited data on strain histogram analysis in itself is available [19–21]. One study on 68 BI-RADS 4 and 5 breast tumours showed slightly lower AUROCs of strain histograms than strain ratios, but direct statistical comparison was not performed [22].

Meta-analyses have shown that both strain and shear-wave elastography perform well in breast cancer diagnosis [6, 23, 24]. As ultrasound BI-RADS scoring has a higher sensitivity than elastography, elastography should be used in conjunction with B-mode imaging only.

Strain ratios provide a semi-quantitative measure of tumour stiffness, which is desirable for malignancy risk stratifications [7, 8]. Reported cut-offs for strain ratios in breast cancer diagnosis vary, probably because strain ratio calculations are affected by placing of reference ROIs and are usually performed on single elastogram frames [7, 9, 25]. Most studies of quantitative elastography such as acoustic radiation force imaging and shear wave elastography use multiple measurements of tumour stiffness for each tumour, which may be time consuming [11, 26]. Also shear-wave speed measurements can be erroneous if transducer precompression is excessive [5, 27, 28]. Precompression is not a confounder in strain elastography, as tumour stiffness evaluation is relative to the surrounding tissue, and both tumour and surrounding tissue are exposed to the same amount of precompression. If implemented in the US-scanner software, strain histogram values can be quickly measured from a single ROI and averaged over an entire elastography cine loop.

When combining a new diagnostic test with an established one, such as the BI-RADS classification, a key matter to consider is how to combine the two tests. Studies have used reclassifications of either BI-RADS 3 or the BI-RADS 4a subclass (low suspicion of malignancy), or both, according to elastography assessments, as these classes have the most false negative and false positive results [11, 12, 17, 18].

For strain ratio calculation, the two ROIs are subject to equal scanning conditions and are therefore theoretically unaffected by anything but the difference in tissue strain. As strain histograms are calculated from a single ROI, tumour depth and size, and breast density could have an impact on strain histogram values. In this study neither tumour depth or size, nor breast density had an impact on strain histograms and strain ratios. In agreement with this, phantom studies have shown, that lesion size carries little impact in strain histogram and strain ratio values [10, 29]. Although an increase in breast density could be thought to increase the stiffness of tissue surrounding breast tumours, this study found no correlation between the two. Previous findings on breast density impact on strain elastography imaging are not unanimous, while no previous studies of lesion depth have been reported [9, 12].

### Limitations

Four out of 26 BI-RADS 3 tumours were malignant which is a high fraction of malignant BI-RADS 3 tumours compared to other studies, which may be due to a small sample size. Larger studies are needed to further clarify the reason for this high malignancy rate. The sensitivity and AUROC for BI-RADS scoring found in this study is comparable to the ones reported in previous work, and specificity was relatively high, signifying that the overall BI-RADS classification was performed adequately [30]. A larger study, with more BI-RADS 3 tumours would make the applicability of strain elastography for this subclass more reliable.

Many studies of ultrasound elastography in breast tumour diagnosis use only histological biopsy as gold standard. In the Scandinavian countries there has been a long tradition of using triple testing in breast diagnosis consisting of palpation, imaging (mammography and/or ultrasound) and fine-needle aspiration in suspicious tumours. Studies have shown that triple testing is equally certain to histological biopsy. We chose therefore to include tumours on which triple testing was used as gold standard.

Strain histogram analysis was performed off-line in this study, which makes the analysis time-consuming and unfit for clinical use. Strain histogram software is however available for some ultrasound systems and should be easy to implement in a clinical set-up.

The study was performed in a specialized breast radiology unit, with a high frequency of malignancies. A strength of the study was that only one, highly experienced breast radiologist performed all examinations, but the results could differ for less experienced operators in less specialized departments. It is a limitation of this study that no inter- or intraobserver evaluation of strain histogram analysis was undertaken. Previous studies have found that both intra- and interobserver agreement is an issue with both semi-quantitative and qualitative strain elastography while shear-wave elastography generally shows less variability [31–33]. To reduce the variability between strain ratios and strain histograms these were performed using similar tumour ROIs.

### Conclusion

Strain histogram analyses are reliable and easy to do in breast cancer diagnosis and perform comparably to strain ratio analyses. Strain histograms and ratios are unaffected by tumour size and depth, and breast density assessed by mammography. No significant difference in AUROCs between BI-RADS scoring and elastography combined with BI-RADS scoring was found in this study.

## Abbreviations

AUROC: Area-under-the-receiver-operating-characteristic-curve
BI-RADS: Breast Imaging Reporting and Data System
ROI: Region of interest
